# Twenty-five years: The fexofenadine clinical experience^☆^

**DOI:** 10.1016/j.waojou.2024.100950

**Published:** 2024-08-20

**Authors:** Robert M. Naclerio, Ignacio J. Ansotegui, Giorgio Walter Canonica, Philip Rouadi, Luo Zhang, Margarita Murrieta-Aguttes

**Affiliations:** a John Hopkins School of Medicine, Department of Otolaryngology-Head and Neck Surgery USA; b Hospital Quironsalud Bizkaia, Spain; c Department of Biomedical Sciences, Humanitas University, Via Rita Levi Montalcini 4, 20090 Pieve Emanuele, Milan, Italy; d Asthma & Allergy Unit-IRCCS Humanitas Research Hospital, via Manzoni 56, 20089 Rozzano, Milan, Italy; e Dar Al Shifa Hospital, Hawally, Kuwait; f Department Otolaryngology and Neck Surgery Beijing Tong Ren Hospital, Beijing Institute of Otolaryngology, Beijing, China; g Sanofi, France

**Keywords:** Oral antihistamine, Fexofenadine, Fast, Long-lasting, Non-drowsy, Allergy, Urticaria

## Abstract

Allergic rhinitis (AR) and urticaria affect a sizable portion of the population worldwide, resulting in reduced quality-of-life and productivity and increased healthcare costs.

Fexofenadine (FEX) is a non-sedating second-generation H_1_ antihistamine with pronounced efficacy and a very good safety profile, used for the treatment of allergic diseases. In addition to its antihistaminic properties, FEX also has anti-inflammatory effects. FEX has a wide therapeutic window and is not associated with any sedative effects, even at higher than recommended doses.

There is a need for an integrated management system for AR and urticaria which includes safe and effective treatment options.

An ideal anti-allergic formulation should provide fast relief of symptoms and long-lasting effect without drowsiness. Data from randomized clinical trials show that FEX meets these criteria and is an effective treatment option with a favourable safety profile, improving the quality of life of patients suffering from AR and urticaria.

## Fexofenadine beyond the histamine blockade: 25 years later

Prevalence of AR continues to rise worldwide in the 21st century and varies across the geographical regions (America, 35%; Europe, 35%; Asia, 22%; Africa, 13%; Oceania, 13%). Major causes for the increase in prevalence are attributed to the hygiene hypothesis, global warming, and air pollution. The impact of allergies on quality of life (QoL) can surpass that of diseases commonly perceived as being more "serious" such as diabetes or hypertension.^1^^,^^2^

AR is associated with several co-morbidities (eg, upper respiratory tract infections, conjunctivitis, severe asthma, atopic dermatitis, chronic rhinosinusitis, otitis media) and sequelae (sleep disturbance, inattention and reduced short-term memory, family, social and behavioral problems, mouth breathing, emotional disorders) which lead to decrease in QoL along with its effects on mental and physical health.^3^

AR and allergic rhinoconjunctivitis (ARC) have a significant impact on education and school performance in adolescents.

**Fig. 1:**
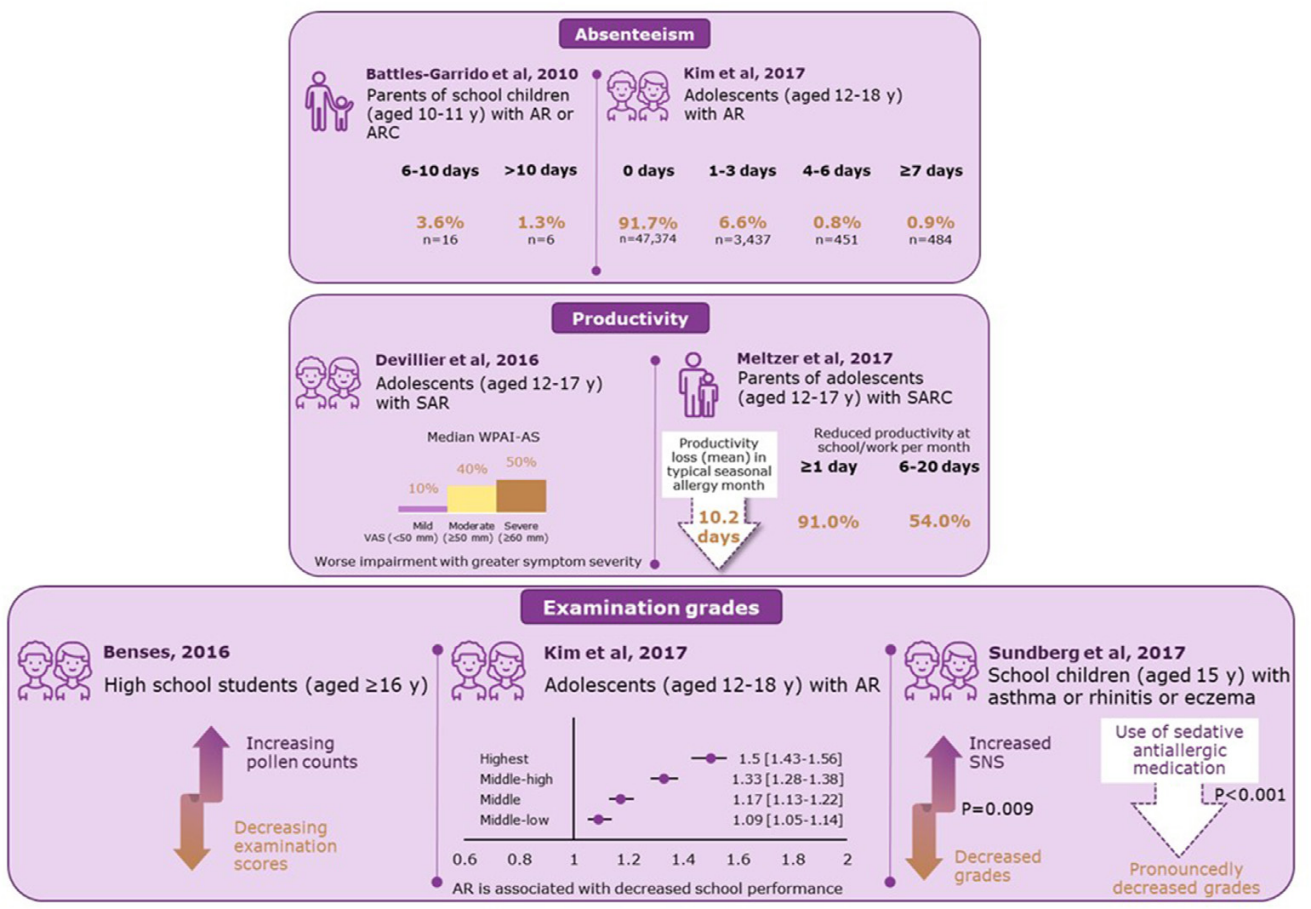
**Effect of allergic rhinitis and allergic rhinoconjunctivitis on education and school performance in adolescents** AR, allergic rhinitis; ARC, allergic rhinoconjunctivitis; CI, confidence interval; OR, odds ratio; QOL, quality of life, RQLQ, rhinoconjunctivitis quality of life questionnaire; SAR, seasonal allergic rhinitis; SARC, seasonal allergic rhinoconjunctivitis; SD, standard deviation; SNS, severe nasal symptoms; VAS, visual analog scale; WPAI-AS, work-productivity and activity impairment questionnaire-allergy specific. Adapted from Sundberg, T et al 2007; Batlles-Garrido, J et al 2010; Bensnes SS 2016; Devillier, P et al 2016; Meltzer, EO et al 2017; Kim SY et al 2017^4^, ^5^, ^6^^,^^8^^,^^10^^,^^12^

A review of literature regarding the burden of AR and ARC in adolescents (aged 10–19 years) found that absenteeism was high in school-going children of age 10–11 years (1–5 days, 21.1%; 6–10 days, 3.6%; >10 days, 1.3%).^4^ In adolescents (12–18 years) with AR, absenteeism of 1–3 days was seen in 6.6% of children, 4–6 days in 0.8% and ≥7 days in 0.9%.^5^ In adolescents (12–17 years) with seasonal allergic rhinitis (SAR), worse impairment of productivity was seen with greater symptom severity. Meltzer et al (2017) reported a mean productivity loss of 10.2 days in adolescents with seasonal allergic rhinoconjunctivitis (SARC) in a typical seasonal allergy month. Among high school children (≥16 years), the examination scores decreased with increasing pollen counts. In school children aged 15 years with asthma or rhinitis or eczema, educational grades were decreased with increased symptoms. Also, grades were pronouncedly decreased with use of sedating antiallergic medication. An improvement in the management of AR led to a significant improvement in academic performance,^5^ In this analysis, South Korean adolescent children (aged 12–18 years) with AR were more likely to report that they achieved the highest level of self-rated academic performance compared with the lowest (Fig. 1).^4^, ^5^, ^6^, ^7^, ^8^, ^9^, ^10^, ^11^, ^12^

*AR significantly impacts exam performance*. A case-control study of students in the United Kingdom sitting national exams in 3 subjects (May–June 2004) found that 36% (662/1834) dropped at least 1 grade in at least 1 subject in summer (pollen season) compared with winter exam. The risk of unexpectedly dropping a grade (cases vs controls) in summer examinations increased after taking first-generation antihistamines (7.7% vs 4.9%; adjusted odds ratio: 1.71, 95% CI: 1.06–2.72) which have a sedating effect and are no longer preferred by physicians.^13^

### Management of AR

Once a diagnosis of AR has been established, the standard of care includes a treatment plan that considers the severity of the disease, the presence of concomitant allergic diseases, and most importantly, a shared decision-making process that focuses on the patient's preferences.

An international, multicenter, cross-sectional epidemiological study conducted in adults and children with AR involving 2778 patients in 11 countries showed that patients prefer to take oral antihistamines (75.9%) and intranasal corticosteroids (49.2%) predominantly, followed by topical decongestants (33.4%), oral decongestants (29.3%) and others.^14^

Second-generation oral antihistamines are fast, long lasting, and well tolerated, ensuring better compliance, whereas intranasal antihistamines have a more rapid onset of action. Addition of antihistamine ± leukotriene receptor antagonist to nasal corticosteroid may be considered as per requirement. Step-up therapy is recommended in case of poor control and step-down therapy if well-controlled. In addition, it is important to avoid triggers. Saline douching and specific immunotherapy may be considered if required.^15^

In case of subliminal allergen exposure, patients may have subclinical inflammation with no symptoms of AR (minimal persistent inflammation). It is important to treat this inflammation.^16^

### Antihistamines

Histamine is an allergic mediator with 3 defined receptors, but the H_1_ receptor is responsible for most of its allergic reactions. Many physicians prefer non-sedating H_1_ antagonists as the initial choice of treatment for AR and urticaria. First-generation antihistamines are associated with multiple side effects due to nonspecific binding to many receptors and penetration of the blood-brain barrier. Unlike first-generation antihistamines, second-generation antihistamines have a better safety and efficacy profile, based on greater potency, receptor specificity, and lower central nervous system penetration.^17^

Treatment with sedating antihistamines in children leads to decreased cognitive and psychomotor abilities, impaired school and/or sport performance/learning, and difficulty concentrating.^18^ Sedating antihistamines are no longer recommended in AR due to lack of good evidence of efficacy and to adverse events (eg, psychomotor retardation and behaviour disturbance).

An ideal antihistamine should be well-tolerated, easy to use, and provide quick relief.^19^ Fexofenadine (FEX) is a non-sedating, second-generation H_1_ antihistamine with great specificity and favourable safety profile.^20^ There is good evidence for the use of FEX in AR, without any psychomotor or behaviour disturbance.

The H_1_ receptor exists in equilibrium with an active and inactive form. Stabilisation of this inactive form shifts the equilibrium towards the inactive state, thereby reducing the number of active receptors to which endogenous histamine may bind.^21^ FEX is an inverse agonist that exhibits an antihistaminic effect by binding the inactive form.^20^^,^^21^ It has been observed that FEX occupies more than 90% of the histamine H_1_ receptors in less than 1 h with a residence time for binding the human H_1_ receptor >100-fold higher than diphenhydramine with a very rapid binding kinetics.^22^

*Effects of FEX on the early response to nasal allergen challenge*

The effects of FEX on the early response to nasal allergen challenge have been shown using different models.

**Fig. 2:**
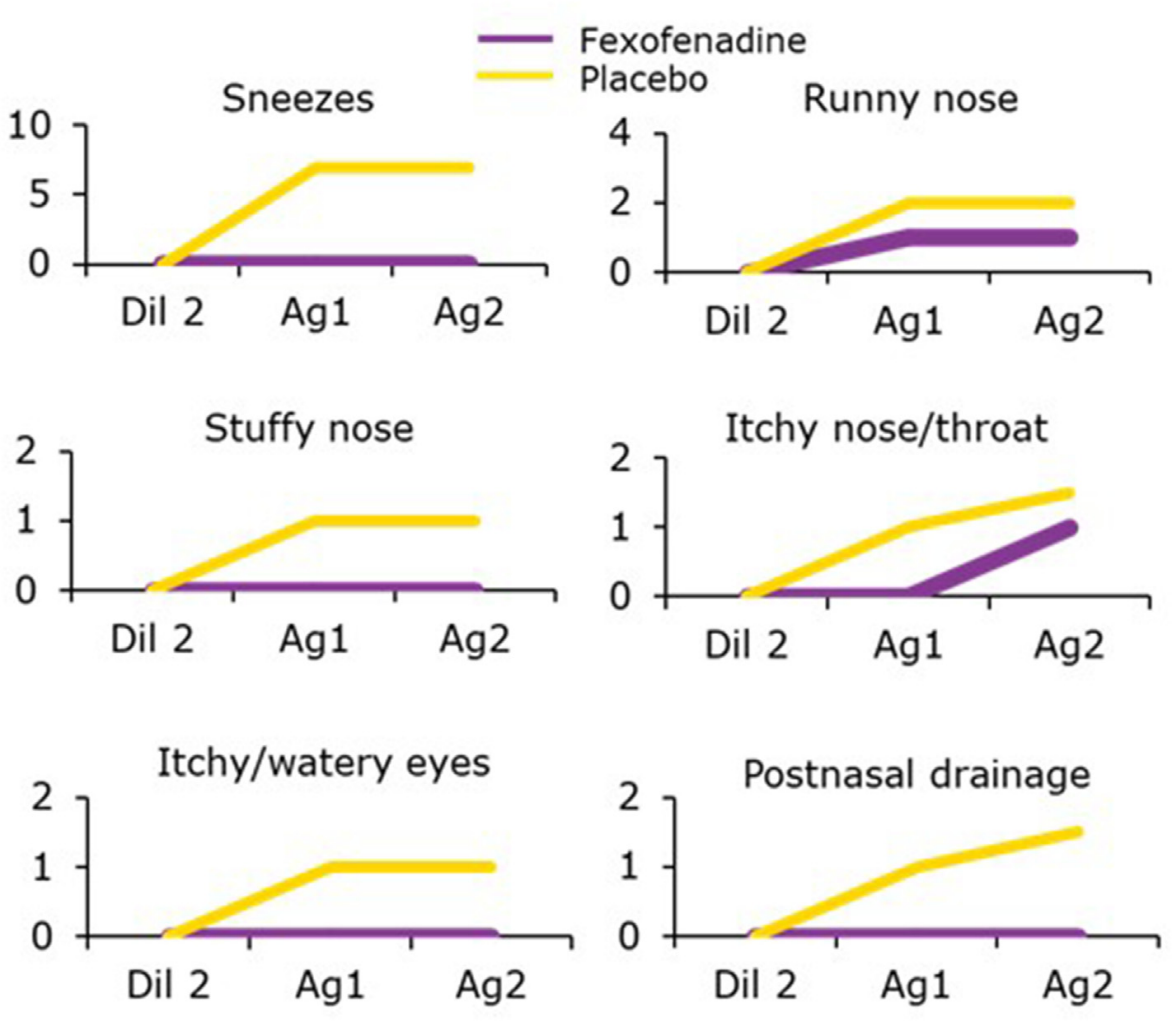
**Effect of fexofenadine on symptoms** Dil2, second diluent challenge; Ag, allergen challenge Adapted from Allocco FT et al, 2002^24^

In a randomized, double-blind, placebo-controlled, two-way crossover study, 20 SAR subjects outside their allergy season received FEX 180 mg once daily (QD) for a week followed by nasal challenge with allergen. FEX inhibited allergen-induced symptoms including nasal congestion and increased vascular permeability but not the release of histamine and tryptase. Pre-treatment with FEX suppressed sneezing, runny nose, stuffy nose, itchy nose/throat, itchy/watery eyes, and postnasal drainage (Fig. 2). This study is a prime example of how pre-treatment works, supporting this concept^23^^,^^24^ These observations are consistent with the hypothesis that the partial reduction of nasal congestion seen with FEX is the result of both its H_1_ blockade and its additional anti-inflammatory effects.^24^

A *histamine-induced inflammatory tissue model*^25^ was used to assess the effect of histamine and its antagonist FEX on fully differentiated primary human nasal epithelia cultured at the air-liquid interface using MucilAir™ material that contains primary nasal cells isolated from 14 different healthy donors. Pre-treatment of nasal tissue with FEX reduced biomarkers of the histamine-induced response (H_1_R, IL-6 and IL-8) versus the condition without pre-treatment confirming that FEX has a dual mode of action, as it inhibited the basal activity of the H_1_R and was more effective reducing biomarkers associated with histamine response when used before and during histamine challenge than when used just during histamine challenge. The effect was dose-dependent regarding H_1_ receptor expression level correlating with inverse agonist activity of FEX.^26^

**Fig. 3:**
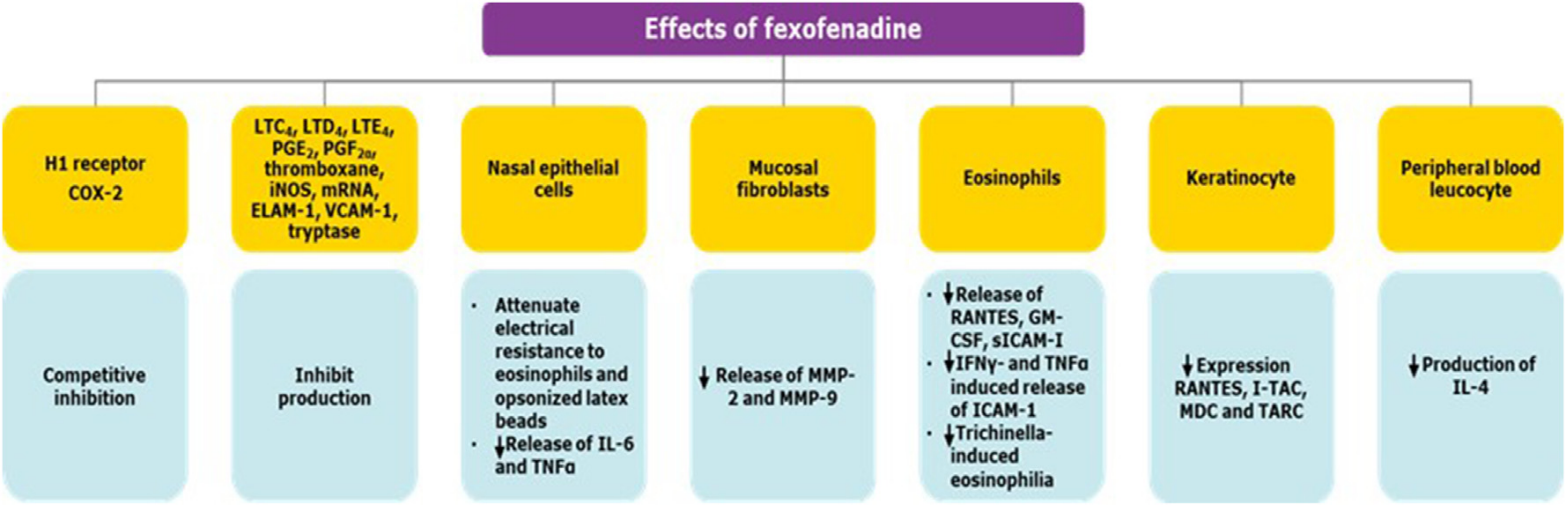
**Mechanism of action of fexofenadine beyond the H**_**1**_**-receptor antagonism** COX-2, cyclooxygenase-2; ELAM-1, endothelial leukocyte adhesion molecule-1; FEX, fexofenadine; GM-CSF, granulocyte-macrophage colony-stimulating factor; ICAM-1, intercellular adhesion molecule 1; IFNγ, interferon gamma; IL, interleukin; iNOS, inducible nitric oxide synthase; I-TAC, interferon-inducible T-cell alpha chemoattractant; LTC4/D4/E4, leukotriene C4/D4/E4; PGE2/F2α, prostaglandin E2/F2 alpha; MDC, macrophage-derived chemokine; MMP-2, matrix metalloproteinase-2; MMP-9, metallopeptidase 9; mRNA, messenger ribonucleic acid; NO, nitric oxide; RANTES, Regulated upon Activation, Normal T cell Expressed and presumably Secreted; sICAM-I, soluble intercellular adhesion molecule-I; TARC, thymus and activation-regulated chemokine; TNFα, tumour necrosis factor alpha; VCAM-1, vascular cell adhesion molecule-1 Adapted from Axelrod D et al., 2008^26^

In addition to antagonizing the H_1_ receptors, FEX decreases the production of LTC_4_, LTD_4_, LTE_4_, PGE_2_, and PGF_2α_; inhibits cyclo-oxygenase 2, the generation of thromboxane (perhaps through cyclo-oxygenase 2); and limits the iNOS generation of NO, as well as the generation of ICAM-1, ELAM-1, VCAM-1, RANTES, I-TAC, MDC, TARC, MMP-2, MMP-9, and tryptase (Fig. 3).^26^

Immuno-histochemical study of non-lesioned skin in patients with active chronic idiopathic urticaria treated with FEX 180 mg QD for 4 weeks showed a prompt and persistent relief of symptoms compared to placebo. In most cases, FEX significantly decreased the expression of Intercellular Adhesion Molecule-1 (ICAM-1) and Endothelial Leukocyte Adhesion Molecule-1 (ELAM-1) on endothelial cells (p < 0.05), decreased the expression of tryptase and some adhesion molecules in urticaria sufferers.^27^

AR is a chronic disease requiring an integrated care for optimal management.

Fexofenadine has been shown effective and well tolerated in a number of randomized, controlled trials (RCTs, see Table 1).^20^^,^^28^, ^29^, ^30^

**Table 1:** Fexofenadine efficacy and safety outcomes from cited randomized controlled trials (RCTs)

Publication	Inclusion criteria	Study population	Treatment arms	Major outcomes
Casale et al.^31^	≥2-y History of moderate/severe SAR, confirmed by positive skin prick test to seasonal allergen	N = 861; 557 women, 304 men; mean age, 32 y (range, 12–85 y)	FEX 120 mg/d for 14 d, FEX 180 mg/d for 14 d, PBO	• Both FEX doses were superior to placebo for reflective TSS assessments (p ≤ 0.0012) • 24-h Reflective nasal congestion scores significantly reduced with FEX 120 mg vs PBO (P < 0.05) • Incidence of adverse events was similar between FEX and PBO groups (30.2% and 30.0%, respectively), with headache the most frequently reported adverse event (8.9% and 7.5%, respectively)
van Cauwenberge and Juniper^29^	≥2-y History of moderate/severe SAR, confirmed by positive skin prick test to grass and/or tree pollen	N = 688; 382 women, 306 men; mean age, 31 y (range, 12–75 y)	FEX 120 mg/d for 14 d, LOR 10 mg/d for 14 d, PBO	• Mean 24-h reflective and instantaneous TSS were significantly reduced by both FEX (both P ≤ 0.0001) and loratadine (P ≤ 0.001 and P ≤ 0.005, respectively) compared with PBO (n = 639) • The incidence of adverse events was low and similar across all treatment groups
Howarth et al.^9^	≥2-y History of moderate/severe SAR, confirmed by positive skin prick test to mixed grass pol	N = 821; 420 men, 401 women; mean age, 33 y (range, 12–66 y)	FEX 120 mg/d for 2 wk, FEX 180 mg/d for 2 wk, CET 10 mg/d for 2 wk, PBO	• There were no differences in efficacy between the 2 doses of FEX or between either dose of FEX and CET. • There was no major side effect, but the combined incidence of drowsiness or fatigue was greater with CET (9%) than with PBO (4%) (P = 0.07) or FEX (4%) (P = 0.02)
Ciprandi et al.^32^	≥2-y History of moderate/severe PAR to dust mite allergen	N = 31; 16 men, 15 women; mean age, 27 y (range, 18–80 y)	FEX 180 mg, 120 mg and PBO QD for 28 days	• Nasal congestion decreased after 1 week of treatment with FEX 120 (P = 0.027) and 180 (P = 0.01), but not with PBO (P = NS)
Okubo K et al.^33^	≥2-y History of PAR, positive nasal provocation test with house dust disc and specific immunoglobulin E (IgE) antibody tests to PAR allergens (i.e., positive to at least one house dust mite, *Dermatophagoides pteronyssinus* or *Dermatophagoides farinae*)	N = 756; Placebo arm: n = 251, 112 males, mean age 34.4 years; Bilastine arm: n = 249, 116 males, 35.9 years; FEX arm: n = 247, 116 males, mean age 36.1 years	Bilastine 20 mg, FEX 60 mg, or PBO (double dummy) administered BID	• Bilastine and FEX showed no significant difference in the primary endpoint.

BID, twice daily; CET, cetirizine; FEX, fexofenadine; LOR, loratadine; PAR, perennial allergic rhinitis; PBO, placebo; QD, once daily; SAR, seasonal allergic rhinitis; TSS, total symptom score

### FEX improves SAR symptoms in children^30^

Children aged 6–11 years (n = 935) received FEX 30 mg BID or placebo for 14 days in a multicentre, placebo-controlled, parallel-group, double-blind study. Symptom scores were significantly improved with FEX. All 12-h-reflective individual symptom scores, including nasal congestion, were significantly reduced compared with placebo (sneezing, p ≤ 0.0001; rhinorrhea, p = 0.0005; itchy nose, palate, throat, and/or ears, p ≤ 0.0001; itchy, watery, red eyes, P ≤ 0.0001; nasal congestion p = 0.0079).

### Efficacy in AR

A metanalysis of 8 double-blind, placebo-controlled randomized-controlled studies found a significant beneficial effect on total nasal symptoms scores and nasal individual symptoms with FEX versus placebo. In patients with SAR, there was a significant beneficial effect with FEX vs placebo on sneezing, nasal itching, nasal congestion, and rhinorrhea (overall effect: −0.27 [p = 0.0006]). No significant differences were found in reports of adverse events between FEX and placebo.^34^

**Fig. 4:**
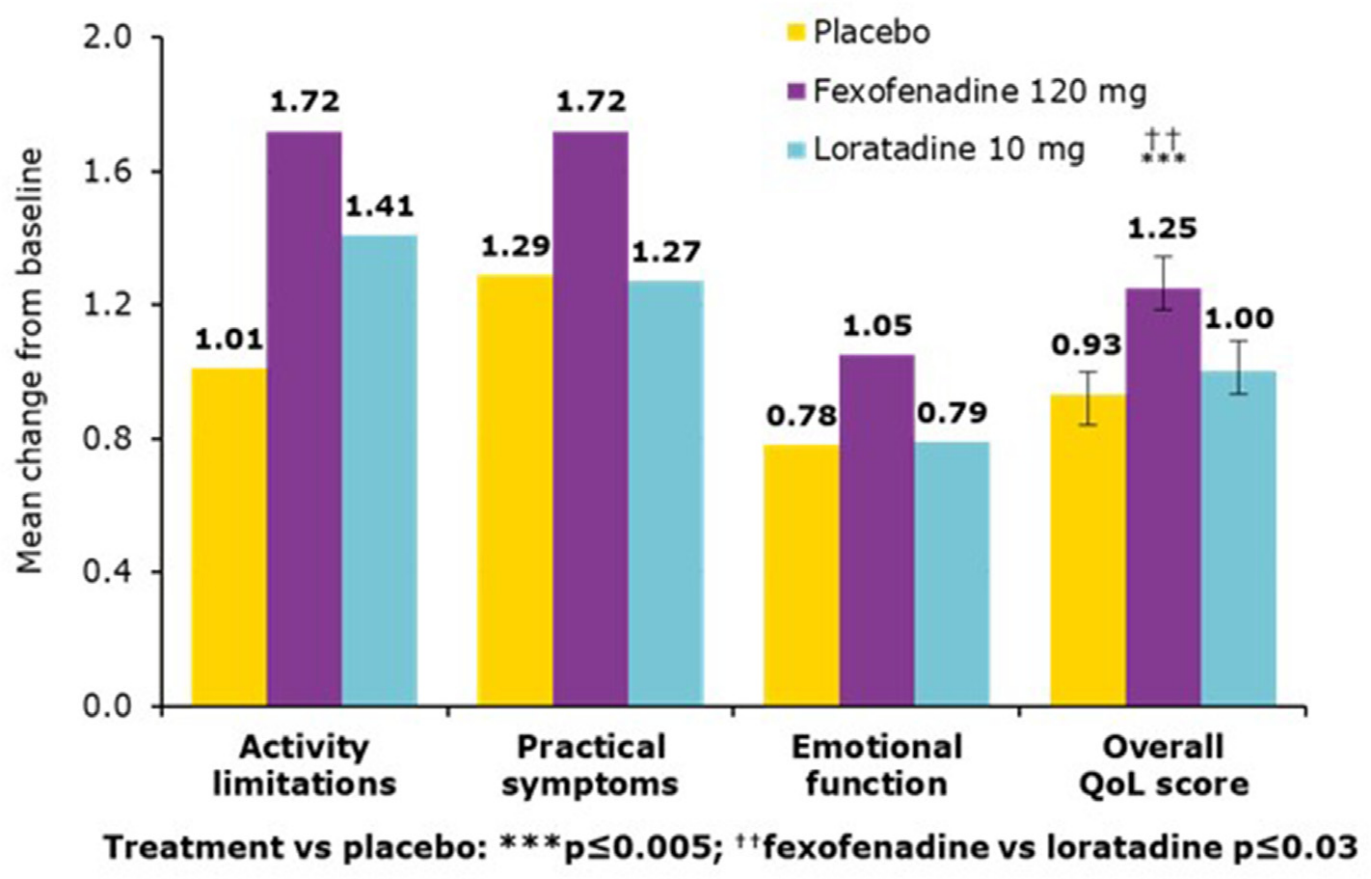
**Mean change from baseline to visit 4 for individual and overall QoL scores**^**a**^ ^a^Data presented as changes in observed means for fexofenadine 120 mg QD, loratadine 10 mg QD and placebo (n = 509). Adapted from van Cauwenberge, P et al. 2008^29^

FEX improves QoL in AR sufferers as demonstrated in a randomized, placebo-controlled study (n = 688), FEX 120 mg QD was significantly superior to loratadine (LOR) 10 mg QD (p ≤ 0.03) and placebo (p ≤ 0.005) in improving QoL (Fig. 4). FEX and LOR significantly reduce the mean 24-h reflective and instantaneous total symptom score (TSS, both p ≤ 0.0001) and LOR (p ≤ 0.001 and p ≤ 0.005, respectively) vs placebo. FEX was significantly better than LOR in improving 24-h reflective itchy, watery, red eyes, as well as relieving nasal congestion (p ≤ 0.05 for both).^29^

In addition, FEX reduces work and activity impairment as shown in a double-blind, placebo-controlled study (n = 845), both FEX 120 mg and 180 mg were found to significantly reduce work and activity impairment vs placebo.^35^

A randomized, double-blind, placebo-controlled, parallel-group, phase III study in 756 Japanese patients with perennial allergic rhinitis showed no significant difference between bilastine 20 mg QD and fexofenadine 60 mg BID in the primary endpoint (Total Nasal Symptom Score from baseline to Week 2).^33^

A multicenter, double-blind, parallel-group, placebo-controlled trial compared the efficacy and safety of FEX (120 and 180 mg administered QD) and cetirizine (10 mg QD) in 722 patients with SAR. There were no differences in efficacy between the 2 doses of FEX or between either dose of FEX and cetirizine.^9^

Patients with AR exposed to pollution and climate change have significant negative impact on health. Epidemiological studies and clinical evidence show the immunological effects after aeroallergen and pollutant co-exposure. Clinical human studies involving specific pollutant exposure and allergen challenge suggest pollution can exacerbate allergic airway disease and increase organ responsiveness.

### FEX reduces SAR symptoms aggravated by air pollutants^36^

**Fig. 5:**
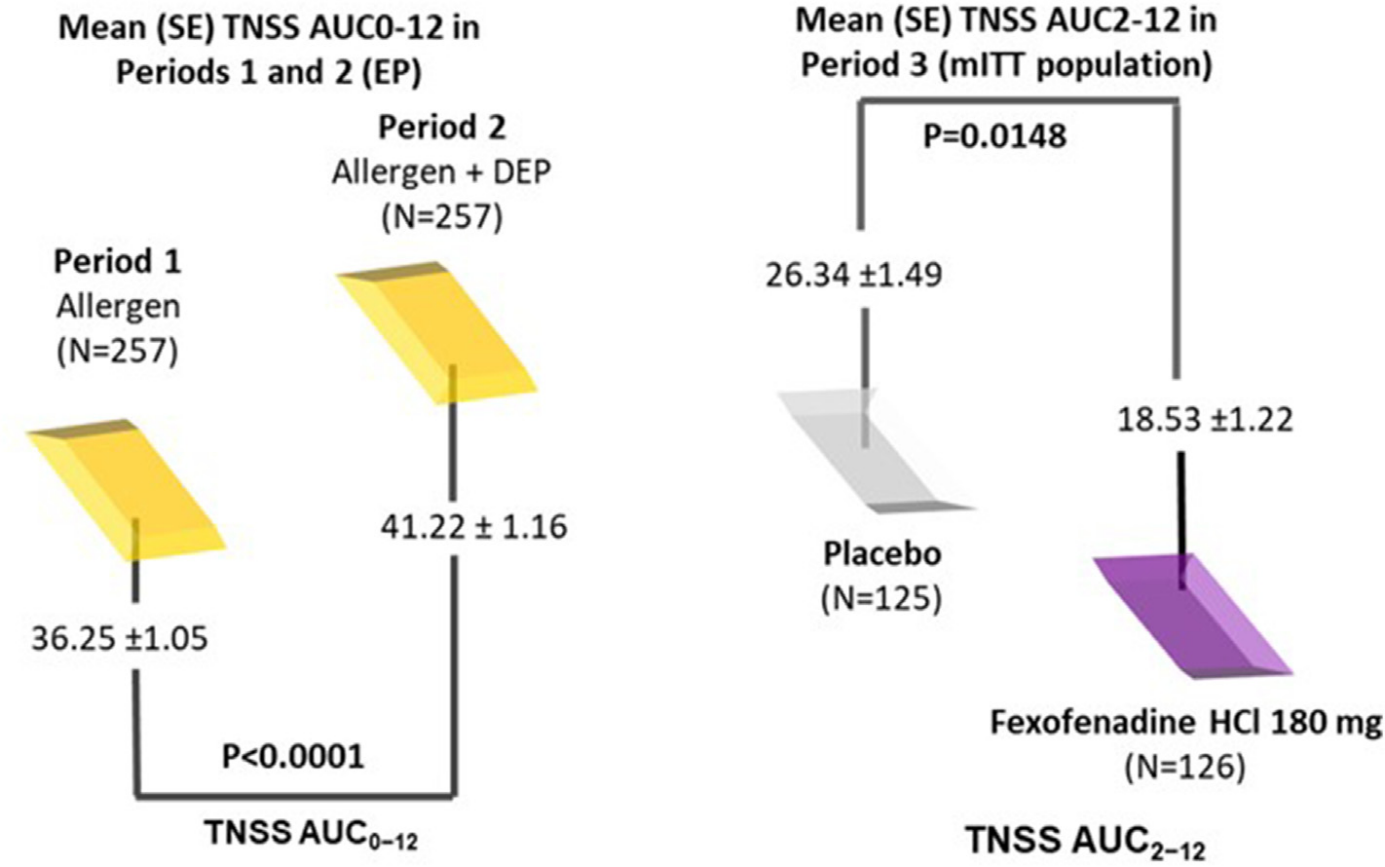
**Mean total nasal symptom score** AUC, area under curve; EP, evaluable population; mITT, modified intention-to-treat; SE, standard error; TNSS, total nasal symptom score Period 1 and 2: AUC time 0–12 h; Period 3: AUC time 2-12 h adapted from Ellis AK et al, 2021^36^

A phase 3, single-centre, sequential, parallel-group, double-blind, randomised study was conducted in an environmental exposure unit (EEU) to assess the efficacy of FEX 180 mg in improving AR symptoms aggravated by air pollutants. Period 1 (ragweed pollen alone), Period 2 [ragweed pollen + diesel exhaust particles (DEP)], and Period 3 (ragweed pollen + DEP + single-dose FEX 180 mg or placebo). Results showed that air pollutant significantly exacerbates SAR symptoms, FEX 180 mg significantly alleviated the pollutant-aggravated symptoms (Total Nasal Symptom Score), and all individual symptoms were improved (Fig. 5).

## An optimal anti-allergic treatment

### Time to onset of symptom relief

In a randomized, placebo-controlled, double-blind, parallel-group study conducted to characterize the time to onset of clinically important relief of AR symptoms in 146 ragweed-sensitive subjects upon treatment with either FEX or placebo, FEX showed the symptom relief in 60 min in 82–85% of patients compared to placebo (p = 0.018).^28^

### Duration of response

In a multicentred, double-blind, parallel-group, placebo-controlled trial in patients with SAR (ITT population = 821; study completed = 722), FEX 120 mg and 180 mg were superior to placebo in reducing the total symptom score. Efficacy was maintained for the entire dosing interval of 24 h.^9^ Similar results have been observed in other RCTs.

### Safety data

It has been demonstrated in a number of RCTs that FEX is not drowsy.^37^, ^38^, ^39^, ^40^, ^41^, ^42^, ^43^ In a randomized double-blind, placebo-controlled, crossover clinical trial, subjective sleepiness and psychomotor performance were measured in 20 healthy volunteers after administration of FEX 120 mg or cetirizine 20 mg.^42^ Higher H_1-_ receptor occupancy in the brain was seen with cetirizine compared to FEX and placebo. In psychomotor tests, FEX was not significantly different from placebo, whereas cetirizine showed a trend towards increased sleepiness compared with FEX and placebo.

In a double-blind, 3-way crossover study with 18 healthy volunteers (20–55 years old) receiving either chlorpheniramine (CPM) 6 mg or FEX 120 mg or placebo QD, CPM 6 mg increased the latencies to sleep onset and rapid eye movement (REM) sleep (p ≤ 0.05 for both), and reduced the duration of REM sleep (p ≤ 0.01).^37^^,^^44^ There were decrements in performance, the next morning (residual effects), with CPM but not with FEX. CPM 6 mg impaired divided attention (p < 0.001), vigilance (p < 0.05), working memory (p < 0.0001) and sensory-motor performance (p < 0.01), and reduced the latency to daytime sleep (p < 0.0001), but not with FEX.

In a randomized, 3-way cross-over, double-blind study of 15 volunteers, evaluating the effect of FEX 360 mg, promethazine 30 mg or placebo in a driving test. No effect on reaction time and critical flicker fusion (CFF) threshold with FEX at 360 mg dose when compared with placebo. If results are extrapolated to real life situation in a motor vehicle being driven at 112 kph, the promethazine would cause the car to travel 3 m extra before the driver engage the brake pedal. Choice reaction time was significantly higher (p < 0.05) with promethazine 30 mg vs FEX 360 mg. At higher doses of 360 mg, FEX does not influence reaction time and CFF threshold when compared to placebo.^41^

In a double-blind, 3-period crossover study, a total of 74 healthy naval flight personnel received either FEX 180 mg or cetirizine 10 mg or placebo. No significant differences between FEX and placebo for any speed measurements under normobaric hypoxic conditions. The number of errors was significantly higher with cetirizine vs placebo (95% CI: 0.0467, 0.3846, p = 0.0127) over the 60 min aeromedical vigilance test and at normobaric hypoxic atmospheric condition. FEX compared to placebo and cetirizine does not cause any increase of risk on the cognitive skills important for piloting.^43^

A multicentre, double-blind, placebo-controlled study evaluated the efficacy of FEX 120 mg or 180 mg vs cetirizine 10 mg or placebo QD in symptomatic patients with SAR showing a similar efficacy with FEX and cetirizine. Incidence of drowsiness and fatigue was similar between placebo and FEX 120 mg or 180 mg. FEX has a comparable frequency of drowsiness/fatigue vs placebo (4% each). Higher combined frequency of drowsiness/fatigue was noted with cetirizine (9%). Adverse events related to study treatment were similar across the treatment groups (23–25%).^10^

Five randomized, multicentre, placebo-controlled studies established the safety and tolerability of FEX in children aged 6 months to 2 y, 2–5 y and 6–11 y old. Minimal difference was observed in the incidence of drowsiness between treatment groups of FEX 15 mg and 30 mg versus placebo among all the age groups evaluated. Similarly, no difference was noted among the study groups when administered as BID dosing.^3^^,^^39^^,^^40^

Based on a large clinical database, fexofenadine HCl had no significant effect on QTc, even at doses >10-fold higher than that is efficacious for AR. Long term studies indicated no statistically significant QTc increases compared with placebo.^45^^,^^46^

### Efficacy in children with SAR

A pooled analysis of 3 double-blind, placebo-controlled studies in pediatric patients (6–11 years) with SAR found that individual nasal and ocular symptoms were significantly improved with FEX vs placebo. Mean change from baseline in the average 12 h-reflective total symptom score was −1.14 for placebo and −1.75 for FEX 30 mg given BID. Safety of FEX was satisfactory and similar to placebo; somnolence was reported in 0.4% of placebo and 0.1% of FEX recipients.^11^

## Chronic urticaria

Urticaria is predominantly a histamine mediated disease. Incidence of chronic urticaria continues to increase in men and women across the world.^47^ A survey in patients with chronic urticaria showed that half of them complained about the effect of their disease on daily functioning (such as sleep, work, school, socializing) and emotions (makes the patient feel annoyed, frustrated, embarrassed, angry, ashamed, anxious, depressed). The results confirmed that chronic urticaria has substantial impact on QoL, with median Skindex-29 scores of 68 for symptoms, 50 for functioning and 53 for emotions.^48^

International societies of allergy coincide in recommending non-sedating second-generation antihistamines as first-line treatment for urticaria.^49^, ^50^, ^51^, ^52^

### FEX significantly improves symptoms of chronic idiopathic urticaria

The efficacy of FEX in the treatment of urticaria has been demonstrated in several RCT.^49^^,^^53^, ^54^, ^55^

**Fig. 6:**
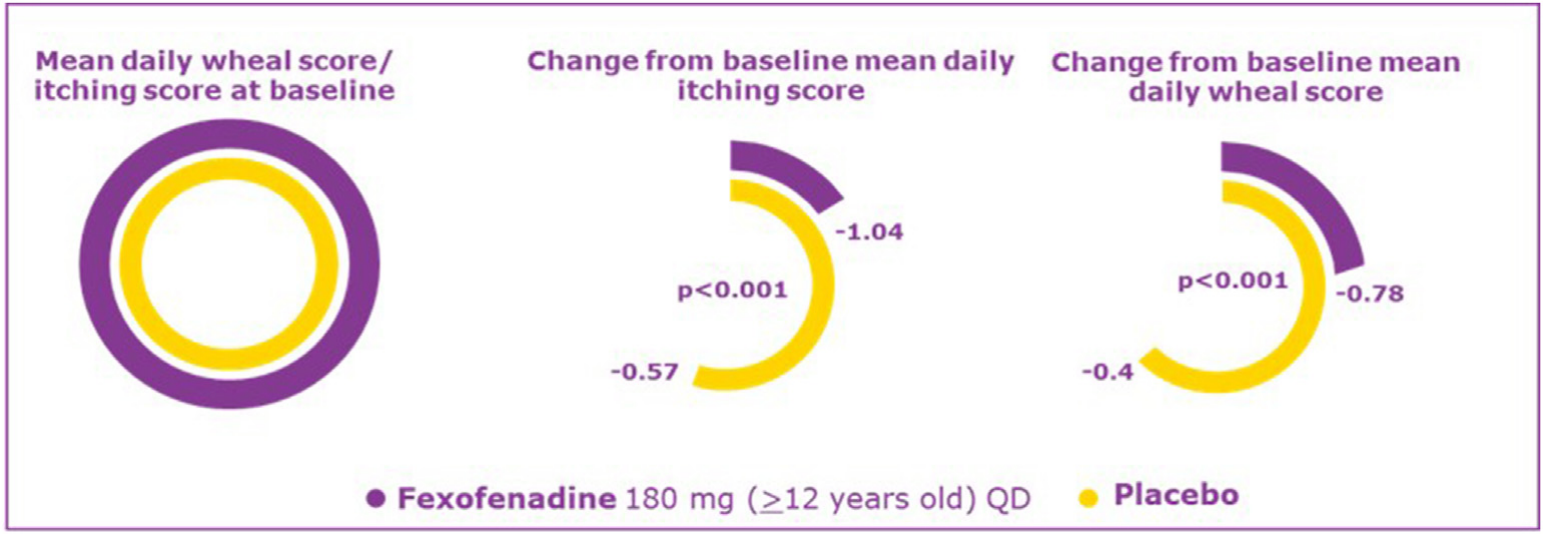
**Reduction of itching and wheals symptoms with fexofenadine treatment** QD, once daily

**Fig. 7:**
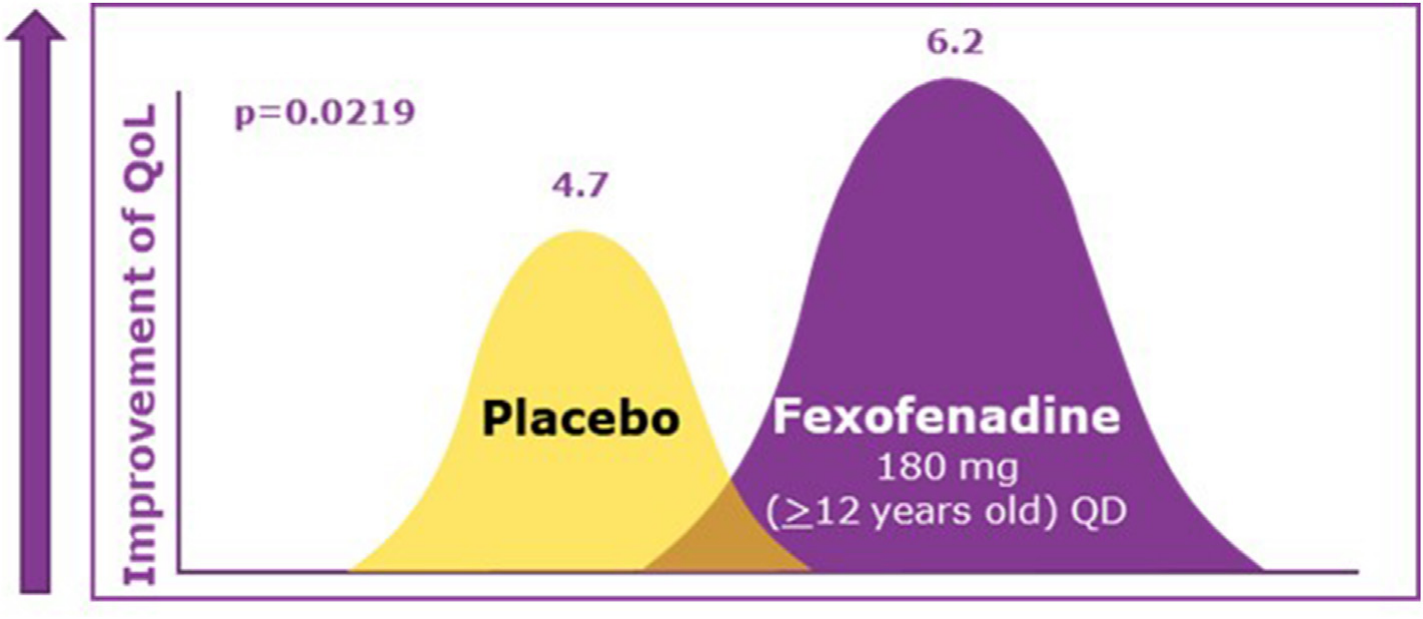
**Improvement in quality of life with fexofenadine treatment in patients with chronic idiopathic urticaria** QD, once daily; QoL, quality of life

A double-blind, placebo-controlled,4-week study in 255 patients with chronic idiopathic urticaria (≥12 y of age) receiving FEX 180 mg once a day showed a significant improvement in QoL indicated by reduced pruritus and wheals in chronic idiopathic urticaria vs placebo (Fig. 6, Fig. 7). In the placebo arm, 37% of patients reported at least 1 adverse event compared to 31% in FEX arm.^53^

In a multicenter, double-blind study, the reduction of mean daily total symptom score of pruritus and wheals was found to be dosage-dependent and statistically significant (p = 0.0041) compared with placebo for the recommended dose of FEX (180 mg).^56^

A randomized, placebo-controlled study enrolling 163 patients (>12 years old) evaluated the mean daily number of wheals and the mean daily severity of pruritus during 180 mg FEX treatment. After a 4-week treatment period, FEX showed greater and significant improvements in both endpoints compared with placebo (mean change in daily number wheals: FEX, −0.78; placebo, −0.40; mean change in mean pruritus severity: FEX, −1.04; placebo, −0.57; p < 0.001 both).^53^

FEX possesses a very good safety profile, with a wide therapeutic window, a minimally effective plasma concentration of ∼15 ng/ml (corresponding to 40 mg daily which is one-third of the recommended dose) and established safety at steady-state plasma concentrations — up to 4677 ng/ml (free from CNS adverse events when assessed objectively at 3 times the recommended dose [360 mg daily: off label] and free from subjective reporting of sedation at 690 mg BID [12x recommended dose: off label]).^54^^,^^55^

A meta-analysis of 8 randomized, double-blind, clinical trials including a total of 3532 participants assessed the efficacy of fexofenadine in AR using adverse events, TSS, and other individual symptom scores as a clinical end point. The safety analysis did not show a significant difference in reported adverse events between the active and placebo treatment groups (p = 0.75).^34^

As previously mentioned, in clinical studies using objective and subjective impairment tests (n = 85) assessing both cognitive and psychomotor performance and feelings of sedation, the effects of FEX were not distinguishable from placebo on a number of tests and have not been associated with any sedative effects, even at higher doses, whereas promethazine caused an overall reduction in CFF thresholds and a significantly higher subjective ratings of sedation when compared to placebo (*P* < 0.05).^41^

In two randomized, double-blind, parallel-group trials of 2-week duration, mean QTc were similar between FEX and placebo in adults and children over a wide range of FEX doses.^39^^,^^45^

## Discussion

FEX is classified as a non-brain-penetrating antihistamines based on the brain H_1_ receptor occupancy (H_1_RO) which is an index of sedative properties.^57^ A review focussed on non-sedative properties of antihistamines for allergic rhinitis treatment summarized that non-brain-penetrating antihistamines like FEX should be considered for the first-line therapy of allergic rhinitis.^57^ FEX is not sedating as demonstrated in a number of randomized, placebo-controlled clinical studies using objective tests.^39^, ^40^, ^41^, ^42^, ^43^

Results of a double-blind, randomized, parallel group, placebo-controlled study shows FEX improves AR symptoms aggravated by air pollutant and may be used for management of AR symptoms aggravated by air pollution.^39^^,^^58^ Second-generation non-sedating antihistamines are the first-line pharmacological approach to resolve urticaria symptoms. FEX is one of the second-generation antihistamines available over the counter and a valid option for the treatment of urticaria in adult and pediatric populations.^49^ A review of the cardiac safety of second-generation H_1_-antihistamines like bilastine, cetirizine, levocetirizine, ebastine, FEX, loratadine, desloratadine, mizolastine and rupatadine found that all these drugs had no evidence of cardiotoxicity even when dosed up to 4 times their standard licensed dose in chronic spontaneous urticaria (off label).^59^ FEX has been found to be free of sedative effects even at higher than therapeutic doses.^60^^,^^61^ FEX improves nasal congestion symptoms more effectively than loratadine. Effect on nasal congestion might be related to its antiallergic effects. A review including nasal challenge studies and clinical trials reported the effects on nasal congestion of the newer second-generation antihistamines desloratadine, fexofenadine, and levocetirizine, showed that in 4 trials reporting objective and/or subjective measures, FEX showed significantly lower nasal congestion scores compared with placebo (P < -0.05).^62^

Inability to cross the blood–brain barrier and high selectivity for peripheral H_1_-receptors might explain the fact that, at even very high doses (360 mg), FEX does not cause sedation and does not impair driving performance. Fexofenadine is not associated with serious cardiac adverse events, and changes in electrocardiogram parameters are not significantly different from those observed with placebo. The high selectivity of FEX for peripheral H_1_-receptors and the lack of interaction with muscarinic receptors might offer a potential advantage compared with other second-generation antihistamines.^63^ An evidence based review of second-generation H_1_-antihistamines in patients with chronic urticaria found that patients who received FEX experienced less work productivity impairment, overall work impairment, and activity impairment than those who received placebo as assessed by the Work Productivity and Activity Impairment (WPAI) questionnaire. In all doses studied, there were no differences in adverse effects between FEX and placebo. Overall, the evidence is high for FEX being well tolerated and effective in chronic urticaria, leading to a strong recommendation for its use in this indication.^64^

## Conclusion

FEX is a non-sedating H_1_ antihistamine with pronounced efficacy and a very good safety profile in the AR and urticaria control improving the patient's QOL. Its efficacy is not just confined to its high affinity towards the H_1_ receptor but may also apply to its anti-inflammatory properties. Second-generation non-sedating antihistamines are first-line therapies for AR and urticaria. Data from RCT showed fexofenadine meets all criteria considered for an optimal allergic disease treatment.

## Abbreviations

AR, allergic rhinitis; ARC, allergic rhinoconjunctivitis; BID, twice daily; CFF, critical flicker fusion; CI, confidence interval; CIU, chronic idiopathic urticaria; COX-2, cyclooxygenase-2; ELAM-1, endothelial leukocyte adhesion molecule-1; FEX, fexofenadine; GM-CSF, granulocyte-macrophage colony-stimulating factor; H_1_R; histamine receptor; ICAM-1, intercellular adhesion molecule 1; IFNγ, interferon gamma; IL, interleukin; iNOS, inducible nitric oxide synthase; I-TAC, interferon-inducible T-cell alpha chemoattractant; LTC4/D4/E4, leukotriene C4/D4/E4; PGE2/F2α, prostaglandin E2/F2 alpha; MDC, macrophage-derived chemokine; MMP-2, matrix metalloproteinase-2; MMP-9, metallopeptidase 9; mRNA, messenger ribonucleic acid; NO, nitric oxide; OR, odds ratio; QD, once daily; QOL, quality of life, QT_c_, corrected QT interval; RANTES, Regulated upon Activation, Normal T cell Expressed and presumably Secreted; RCT, randomized controlled trials; RQLQ, rhinoconjunctivitis quality of life questionnaire; SAR, seasonal allergic rhinitis; SARC, seasonal allergic rhinoconjunctivitis; SD, standard deviation; sICAM-I, soluble intercellular adhesion molecule-I; SNS, severe nasal symptoms; TARC, thymus and activation-regulated chemokine; TNFα, tumour necrosis factor alpha; VAS, visual analog scale; VCAM-1, vascular cell adhesion molecule-1; WPAI-AS, work-productivity and activity impairment questionnaire-allergy specific.

## Funding

Not applicable.

## Availability of data and materials

Data sharing is not applicable to this article as no datasets were generated or analyzed during the current study.

## Author contributions

All authors were involved in the conception of the work and critically revising the manuscript, and take full accountability for the work, for all content, and editorial decisions. All authors approved the final version to be published.

## Ethics approval

This article is based on previously conducted studies and does not contain any new data collected from human participants or animals.

## Consent for publication

All named authors have given their approval for this manuscript to be published.

## Declaration of competing interest

Luo Zhang, MD and Girgio Walter Canonica, MD have no relevant disclosures. Robert M. Naclerio, MD has received speaker fees from Sanofi and Regeneron and is a member of an advisory board for Lyra. Ignacio J. Ansotegui, MD has received fees from Bayer, Bial, Eurodrug, Faes Farma, Menarini, MSD, Roxall and Sanofi. Philip Rouadi, MD has received speaker fees from Sanofi, MSD, and Menarini foundation. Margarita Murrieta-Aguttes, MD is an employee of Sanofi.
